# A PKC-Dependent Recruitment of MMP-2 Controls Semaphorin-3A Growth-Promoting Effect in Cortical Dendrites

**DOI:** 10.1371/journal.pone.0005099

**Published:** 2009-04-08

**Authors:** Bertrand Gonthier, Eric Koncina, Saulius Satkauskas, Martine Perraut, Guy Roussel, Dominique Aunis, Josef P. Kapfhammer, Dominique Bagnard

**Affiliations:** 1 INSERM U575, Physiopathologie du Système Nerveux, Strasbourg, France; 2 Vytautas Magnus University, Department of Biology, Kaunas, Lithuania; 3 Developmental Neurobiology, Institute of Anatomy, University of Basel, Basel, Switzerland; Tufts University, United States of America

## Abstract

There is increasing evidence for a crucial role of proteases and metalloproteinases during axon growth and guidance. In this context, we recently described a functional link between the chemoattractive Sema3C and Matrix metalloproteinase 3 (MMP3). Here, we provide data demonstrating the involvement of MMP-2 to trigger the growth-promoting effect of Sema3A in cortical dendrites. The in situ analysis of MMP-2 expression and activity is consistent with a functional growth assay demonstrating in vitro that the pharmacological inhibition of MMP-2 reduces the growth of cortical dendrites in response to Sema3A. Hence, our results suggest that the selective recruitment and activation of MMP-2 in response to Sema3A requires a PKC alpha dependent mechanism. Altogether, we provide a second set of data supporting MMPs as effectors of the growth-promoting effects of semaphorins, and we identify the potential signalling pathway involved.

## Introduction

Several families of guidance molecules including eph/ephrins [1], [2], netrins [3], slits [4] and semaphorins [5], [6] have been identified over the past ten years. These secreted or membrane anchored signals have growth promoting or growth inhibitory properties leading to the appropriate orientation of process outgrowth [7]. The mechanisms initially described for axon guidance have now been shown also to control dendritic growth and guidance [8] consistently with the many roles described for semaphorins [9]. This is particularly the case for class 3 semaphorins which are key regulators of cortical wiring [10], [11]. Once they reach their laminar position in the nascent cortical plate, layer V and VI cortical neurons extend long axonal projections to the thalamus or the spinal cord. Gradients of Sema3A are thought to repel these cortical efferent fibers away from the ventricular zone to reach the internal capsule [10], [11]. Surprisingly, Sema3A has also been shown to control the dendritic development of cortical neurons. This unexpected effect is particularly striking because Sema3A acts on dendrites as a growth promoter instead of triggering its classical inhibitory effect [12], [13]. The dual function of Sema3A in cortical neurons appears to be linked to sub-cellular differences in cGMP localization [12]. The soluble form of the adhesion molecule L1 has also been shown to convert the chemorepulsive effect of Sema3A into a chemoattractive one [14]. The most detailed pathway of the growth promoting effect of semaphorins has been recently obtained during the molecular dissection of the Sema3B-dependent positioning of the anterior commissure. In this study the selective recruitment of the focal adhesion kinase (FAK) and the activation of the Src kinase family were shown to define the attractive effect of Sema3B [15]. Nevertheless, the exact nature of the signalling cascade encoding the growth promoting activity of semaphorins remains obscure. In search for signalling elements of the semaphorin growth promoting pathways, we recently identified a functional interaction between Sema3C and matrix metalloproteinases (MMPs) [6]. The MMPs are proteolytic enzymes ensuring various functions ranging from cell proliferation and migration to cell surface receptor cleavage [16]. Our results demonstrated that MMP-3 (stromelysin-1) is expressed and activated in growing cortical axons. Strikingly, Sema3C increases both the expression and activity of MMP-3, and the chemoattractive effect of Sema3C is abolished by a specific inhibitor of MMP-3. The chemorepulsive Sema3A was shown to reduce MMP-3 expression and activity consistently with its inhibitory effect on axons. Thus, similarly to what has been described for ephrins [17] and netrins [18], a metalloproteinase activity is required to achieve the appropriate signalling of semaphorins. To characterize further the role of MMPs during corticogenesis and semaphorin signalling we decided to search for a role of MMPs in the chemoattractive effect of Sema3A on cortical neuron dendrites. Our results demonstrate that the growth promoting effect of Sema3A on cortical dendrites requires MMP-2 by a mechanism of transduction implicating at least neuropilin-1 and a PKCα-dependent pathway.

## Results

### A gelatinolytic activity is detected in the developing cortex

**Figure 1:**
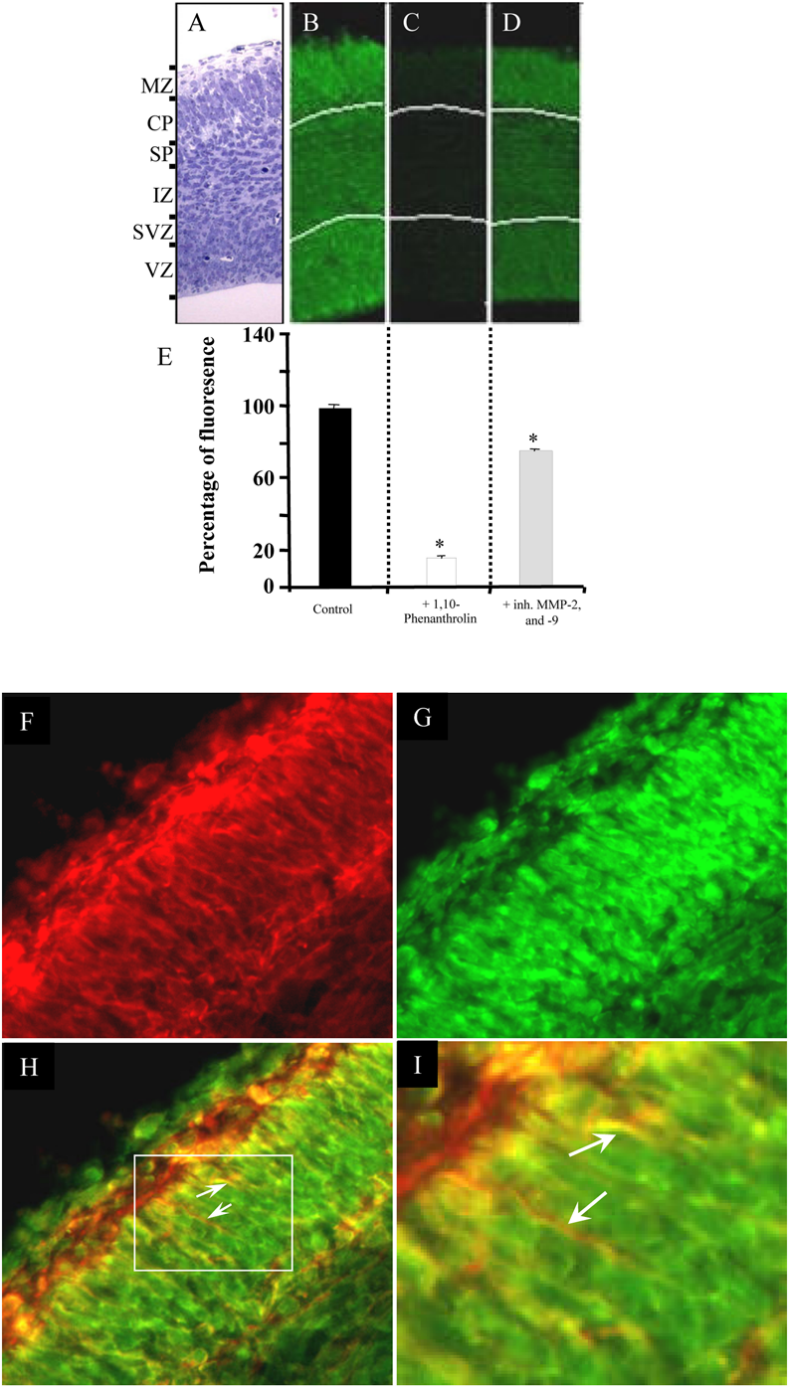
Activity of MMPs in the developing cortex. (A–E) Quantification of the MMP activity revealed by *in situ* zymography performed with FITC-conjugated gelatin. (A) Cytoarchitectural organization of the E15 neocortex visualized by semi thin section of E15 neocortex stained with toluidin blue. (B, C, D) Representative microphotographs of *in situ* zymography performed with FITC-conjugated gelatin in control condition (B), in the presence of 1,10-phenantroline (general MMP inhibitor) (C), and in the presence of a selective inhibitor of MMP-2/-9 (D). (E) Quantification of the zymographic signals in the entire neocortex. (F–H) Co-localization of MMP-dependent gelatinolytic enzymatic activity (viewed by *in situ* zymography) with MAP2 in E15 neocortex. (F) The immunostaining of the dendrites with anti-MAP2, (G) the corresponding zymographic signal and (H) the overlay of the two images. (I) Higher magnification of the white selection box in the overlay. (H, I: white arrow indicates gelatinolytic activity surrounding growing dendrites). MZ: Marginal Zone; CP: Cortical Plate; SP: Subplate zone; IZ: Intermediate Zone; SVZ: Sub-Ventricular Zone; VZ: Ventricular Zone.

To address the potential role of MMPs during development of cortical dendrites we searched for the existence of a gelatinolytic activity in the developing E15 cortex. Using in situ zymography we found a strong activity of gelatinases in the entire neocortex (Figure 1). Both dividing cells of the ventricular zone and differentiated cells of the cortical plates showed a capacity to degrade FITC-conjugated gelatin. We verified the specificity of the signal by addition of ortho-phenantroline that suppresses fluorescent signal. Strikingly, the addition of a MMP-2/9 (Gelatinase A and B) inhibitor induced a significant reduction of the gelatinolytic activity thereby supporting a role of these proteins in the observed global enzymatic activity. The combination of in situ zymography and immunostaining of the dendritic MAP2 marker showed that such a gelatinolytic activity is present at the level of cortical dendrites. This result is consistent with a role of MMPs during cortical development, including dendritogenesis. These data were obtained from collections of 8 different brains from which 5 different slices were analyzed (coronal sections, observation at the telencephalon/diencephalon border).

### Developing cortical dendrites express MMP-2

**Figure 2:**
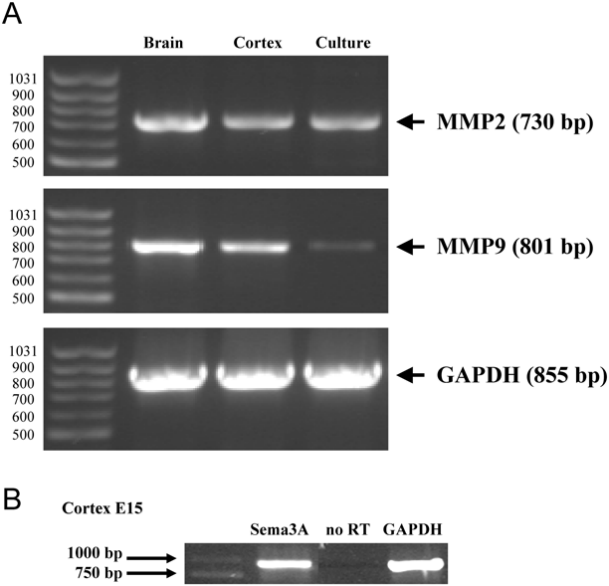
Expression of MMP2 and MMP9 in cortical neurons. (A) RT-PCR analysis of MMP2 and MMP9 transcripts in E15 whole brain, E15 whole cortex and dissociated E15 cortical neurons after 2 day in culture. GAPDH housekeeping gene was used as internal control for each sample (bottom panel). B) RT-PCR analysis of Sema3A transcripts in cortical neurons. Molecular weight marker (GeneRuler™ 100 bp DNA Ladder, Fermentas) was used to check for correct transcript band (on left).

**Figure 3:**
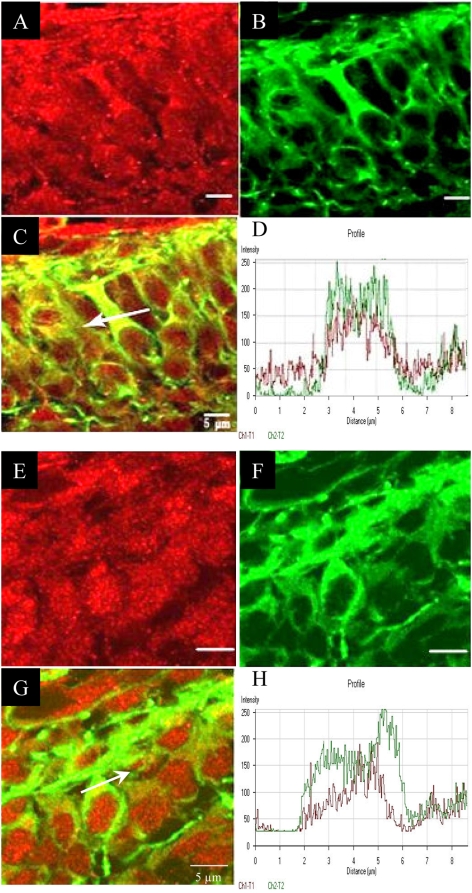
Expression of MMP2 at the apical dendrites of cortical neurons. Expression pattern of MMP-2 and MMP-9 in the mouse neocortex at E15. (A–D) Microphotographs were obtained from confocal microscope analysis of the double staining (C) for MMP-2 (A), and MAP2 (as a dendritic marker) in dendrites growing in the cortical plate (B). (D) Co localization profile of MMP-2 and MAP2 signals demonstrating the presence of MMP-2 in the dendrites. (E–H) Confocal microscope images of the double staining (G) for MMP-9 (E) and MAP2 (F) in dendrites of the cortical plate. (H) Co localization profile of MMP-9 and MAP2. Here the non-superposed graphs prove the absence of co localization between MMP-9 and MAP2. White arrows point out representative line profiles allowing better visualization of double staining at the sub cellular level.

To investigate the potential role of gelatinases during cortical dendrite development we investigated the expression of gelatinases in the developing cortex. Using RT-PCR, we searched for the presence of transcripts of the two classical gelatinases MMP-2 and MMP-9 in the full brain (5 samples), in micro-dissected neocortex (>25 samples) and in cultured cortical neurons (4 different cultures). As depicted in Figure 2 showing the results of representative samples, the two MMPs are expressed in all conditions. We noticed that MMP-9 expression was weaker in cultured neurons. Interestingly, cortical neurons also expressed significant levels of Sema3A mRNA (Figure 2B). To address the cellular expression of MMP-2 and MMP-9, we performed in situ immunocytochemical analyses on samples taken from the collection used for in situ zymography (with a minimum of 5 slices per conditions). Specific antibodies to MMP-2 and MMP-9 in combination with anti-MAP2 antibody allowed us to search for the expression of the two gelatinases in cortical dendrites. As seen in Figure 3, confocal microscopic analysis showed weak staining of MMP-9 in cortical neurons with no obvious co-localisation with the dendritic MAP2 marker. On the other hand, these neurons express a high level of MMP-2, particularly in the apical dendrite. This result is in favour of a role of MMP-2 in cortical neuron dendritic outgrowth. Together with the in situ zymography, this observation prompted us to examine whether MMP-2 expression varied in response to Sema3A.

### The Sema3A-dependent dendritic growth promoting effect is gelatinase-dependent

**Figure 4:**
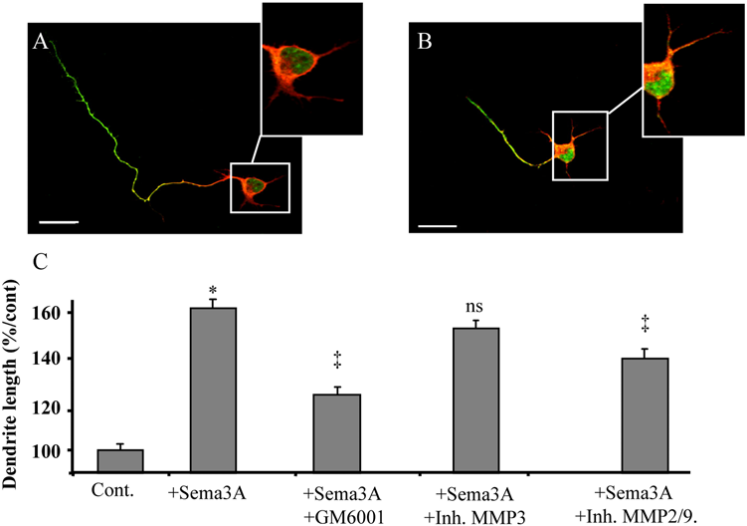
MMP-dependent Sema3A-induced dendritic outgrowth. (A–B) Double staining on dissociated cortical neurons for neurofilament (in green, as an axonal marker) and MAP2 (in red, as a dendritic marker) illustrating the decrease of axonal length and the increase of dendritic length in presence (B) or not (A) of Sema3A. (C) Quantification of the dendritic growth promoting effect of Sema3A. Cortical neurons are grown without treatment or with Sema3A + a large spectrum MMP inhibitor GM6001, or selective inhibitors of MMP-2/9 or MMP-3. (* p<0.01 student's t test, ns not significant).

Some evidences suggest a growth promoting role of Sema3A for cortical neuron dendrites [12]. We decided to culture dissociated cortical neurons in the presence of purified Sema3A in order to quantify this positive effect. The use of a double immunostaining for axonal neurofilament (SMI312) and dendritic MAP2 allowed us to discriminate between the two types of processes as early as 24 hours in culture. As expected, we found that the dendritic length of cortical neurons were 40–60% increased with 100 ng/ml Sema3A. Consistent with previous description of the inhibitory function of Sema3A, this increase of dendritic length was concomitant with a decrease of axonal length (data not shown). To address the potential role of MMPs in the Sema3A-dependent dendritic growth promotion, we used various pharmacological inhibitors of MMPs (Figure 4). We, and others, previously demonstrated the specificity of this strategy. Control experiments (without Sema3A) revealed no change of dendritic length in the presence of MMP inhibitors (see Figure S1). In the other hand, the use of 1 μM GM6001, a large spectrum inhibitor of MMPs, triggered a strong reduction of the Sema3A-growth promoting effect. The growth promoting effect was conserved in the presence of a specific inhibitor of MMP-3 that has been shown to suppress the growth promoting effect of Sema3C for cortical axons [6]. On the other hand, a gelatinase inhibitor (inhibitor of MMP-2 and MMP-9) significantly reduced the dendritic growth promoting effect of Sema3A. Thus, a gelatinase activity is required for the Sema3A growth promoting effect. These data were obtained from 11 independent experiments with a total of 1837 neurons analyzed.

### Sema3A increases expression and activity of MMP-2

**Figure 5:**
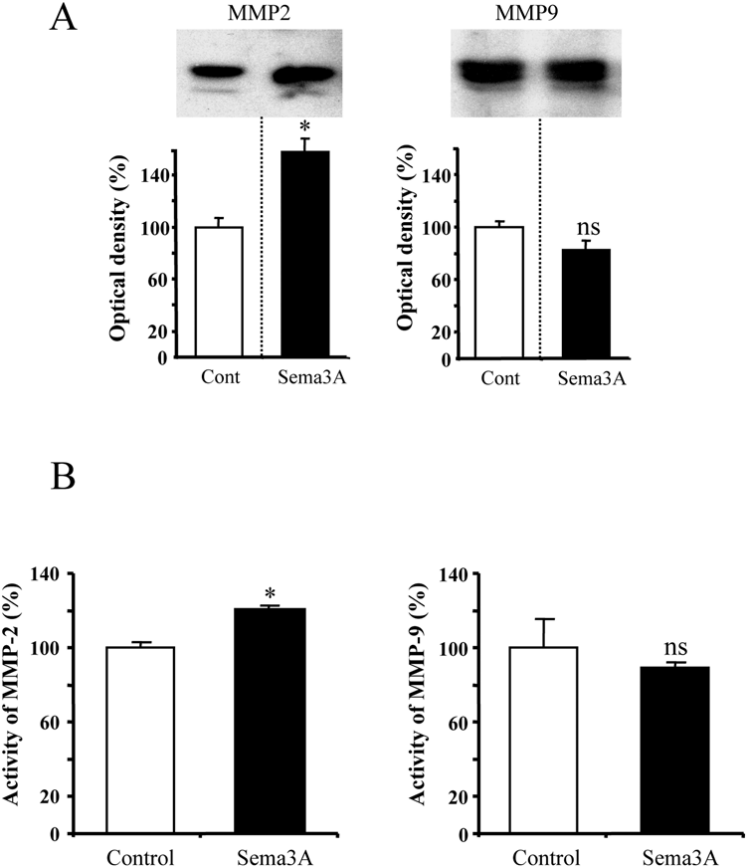
Sema3A-dependent expression and activity of MMP-2. (A) Western Blotting analysis of MMP-2 and MMP-9 expression. The expression of MMP-2 increases in the presence of Sema3A, while no modification occurs for MMP-9. (B) Determination of MMP-2 and MMP-9 enzymatic activity by ELISA assay. MMP-2 activity increases in the presence of Sema3A without modification MMP-9 activity. (* p<0.01 student's t test/control, ns not significant).

**Figure 6:**
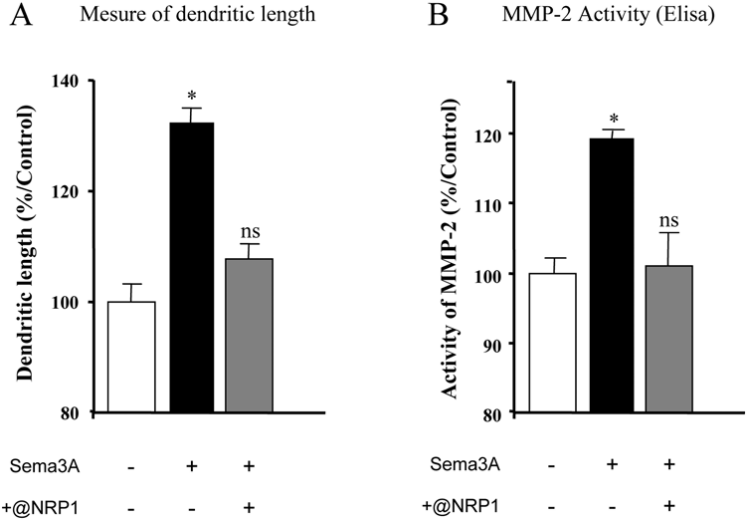
Specificity of Sema3A-induced increase of MMP-2 activity. (A) Dissociated neurons were grown without treatment or in the presence of Sema3A±blocking antibodies. The block of Neuropilin-1 (NRP1) suppressed Sema3A-induced dendritic growth. (B) Quantification of MMP-2 activity in culture media from dissociated neurons treated or not with Sema3A±anti-NRP1. The block of Neuropilin-1 (NRP1) abolished Sema3A-induced increase of MMP-2 activity. (* p<0.01 student's t test/control, ns not significant).

To characterize further the involvement of gelatinases in the growth promoting effect of Sema3A, we performed western blot analysis (4 independent experiments) and enzymatic activity assays (3 independent experiments including triplicates of each experimental condition). As depicted in Figure 5A, cortical neurons in culture express both MMP-2 and MMP-9 consistently with RT-PCR and in situ observations. The addition of Sema3A in the culture medium induced 49% increase of MMP-2 expression while MMP-9 expression remained unchanged. Thus, Sema3A triggers a specific induction of MMP-2 expression. Using an Elisa-based activity assay, we also found that the induction of MMP-2 expression was associated with an increased enzymatic activity (+23%) in culture media while MMP-9 activity was not changed (Figure 5B). These results demonstrate that Sema3A signalling triggers a specific induction of MMP-2 expression and activity. To demonstrate the specificity of the link between MMP-2 and Sema3A we monitored MMP-2 activity in the presence of a function-blocking antibody directed against Neuropilin-1, the major binding receptor of Sema3A. To this end, cortical neurons were grown in control conditions or in the presence of 100 ng/ml Sema3A, with or without 1 μg/ml anti-NRP1. As expected from previous studies, the selective blocking of NRP1 suppressed the Sema3A-dependent growth promotion of cortical dendrites (Figure 6A). Consistently, we found that blocking NRP1 suppressed Sema3A-induced augmentation of MMP-2 activity (Figure 6B).

### Sema3A-induced MMP-2 up-regulation is PKC dependent

**Figure 7:**
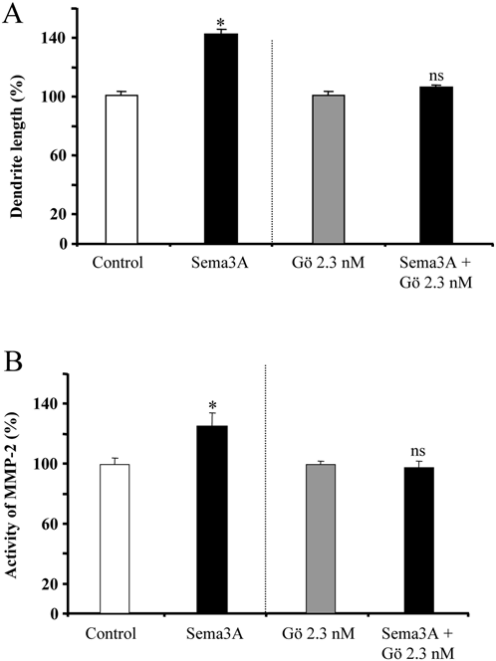
Sema3A growth promoting effect is PKC-dependent. (A) Analysis of embryonic neurons dendritic length. Treatment of dissociated neurons with 100 ng/ml of Sema3A and/or with Gö 6976 increased dendritic length. (B) Analysis of MMP-2 activity by enzymatic ELISA assay. Sema3A treatment increased MMP-2 activity in culture media from cultured neurons. The inhibition of PKCα with 2.3 nM of Gö suppressed the increase of MMP-2 activity induced by Sema3A. (* p<0.01 student's t test/control, ns not significant).

**Figure 8:**
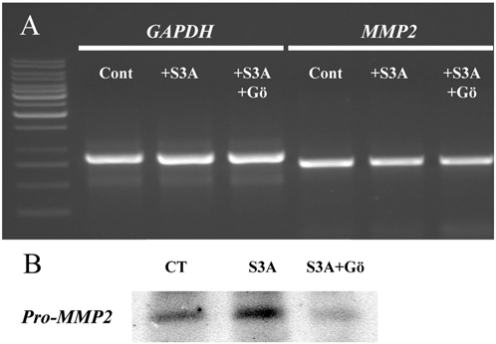
Transcriptional and translational regulation of Sema3A-induced MMP2 expression. The inhibition of PKCα blocks Sema3A-induced MMP-2 at the protein level but not at the transcriptional level. (A) RT-PCR analysis revealing no change of MMP-2 expression at the transcription level. (B) Western blot analysis revealing the lack of Sema3A-induced MMP-2 protein level augmentation in the presence of Gö 6976 (translational effect).

Recent evidence suggests that semaphorins and MMPs share common intracellular pathways. This has been shown for PKC, a central regulator of signalling cascades [19]. To address the question of the signalling mechanism linking Sema3A to MMP-2 we cultured neurons in the presence of the potent PKC inhibitor Gö6976, which is supposed to be specific for the classical Ca^2+^-dependent isoforms. As illustrated in Figure 7A, the growth promoting effect of Sema3A was abolished in cortical dendrites by 2.3 nM Gö. Moreover, Sema3A-dependent activation of MMP2 is abolished in the presence 2.3 nM Gö. A western blot analysis showed that PKC inhibition also blocked Sema3A-induced MMP2 expression (Figure 8B, 3 independent experiments). No change occurred at the transcriptional level (Figure 8A, 4 independent experiments). Considering the activity spectrum of Gö6976, our results are consistent with a role of PKC alpha to trigger the growth promoting cascade of Sema3A. Thus, the selective recruitment of MMP-2 to trigger Sema3A dendrite growth promotion requires PKCα activation.

## Discussion

Sema3A has been described as a chemottractant for the dendrites of cortical neurons, a surprising effect in comparison to the inhibitory effect of Sema3A in axons [12]. The molecular mechanisms mediating this effect remain largely unknown. Based on our previous finding of a role of MMP-3 in the growth and guidance of cortical axons in response to the chemoattractive Sema3C, we decided to investigate whether in the positive semaphorin signalling, members of the MMP family would also be involved. Our results provide evidence for a role of MMP-2 to trigger the growth promoting effect of Sema3A. Because cortical neurons express Sema3A, this mechanism is potentially cell autonomous.

This result reinforces our previous observation suggesting a wide implication of MMPs in the development of embryonic brain [6]. The use of in situ zymography showed that a gelatinolytic activity is present in the entire thickness of the neocortex in dividing, migrating or differentiating cells. The exact functions of MMPs will have to be clarified in future studies. In the present study, we focused on the proteolytic activity observed at the level of growing dendrites. Unfortunately, no selective pharmacological inhibitors were available to discriminate between MMP-2 and MMP-9, the two major gelatinases. The one we used in our experiments blocks completely MMP-2 and MMP-9 activity. In this case, the Sema3A growth promoting effect on dendrites was only partially suppressed suggesting that these two MMPs are not the only ones to be involved. This was confirmed through the use of the large spectrum MMP inhibitor GM6001 which was found to produce a stronger reduction of the growth promoting effect of Sema3A. Western blot analysis and ELISA-based activity assay using specific antibodies against MMP-2 or MMP-9 showed that both protein level and activity of MMP-2 but not MMP-9 were increased in response to Sema3A. This is consistent with the strong expression of MMP-2 in developing dendrites find here to co localize with MAP2. A recent report also showed dendritic expression of MMP-2 in E18 cortical dendrites [20]. In our model, MMP-9 has no obvious effect. This contrasts with the results obtained in the adult mice showing the involvement of MMP-9 for dendritic remodelling following kaïnate injection in the hippocampus [21]. MMP-9 is also known to regulate axon growth in the cerebellum [22], [23]. Taken together, these and our results indicate that MMPs elicit cell-type and time-dependent biological functions in the developing and mature brain. This is consistent with the increasing demonstration of ADAM function in axon guidance [24].

The receptor complex involved in the Sema3A-dependent growth promotion has still to be clearly characterized. Our data suggest that Sema3A-induced cortical dendrite outgrowth is NRP1-dependent. The involvement of NRP1 is not surprising because this receptor is the preferential binding partner of Sema3A [25]–[28]. However, the composition of the receptor complex allowing NRP1 to induce a growth promoting effect is not known. In the zebrafish, the binding of Sema3D on a homodimer of NRP1 induces repulsion while the binding of Sema3D on a heterodimer composed of NRP1 and NRP2 induces attraction [29]. We also have shown that the Sema3C-chemoattractive effect on cortical axons requires NRP2 [6]. The exact role of NRP1 and NRP2 homo and heterodimers has to be clarified in this context. In any case, the functional link we discovered between MMP-2 and Sema3A provides the second set of data supporting the involvement of matrix metalloproteinase in semaphorin signalling. Thus, as for netrins [18], ephrins [17], [30] or slit [31], a protease activity appears to be required to trigger appropriate guidance cues signalling pathways, hence placing proteolytic activity as a key actor of nervous system wiring. This is consistent with recent data that showed in vivo the importance of MMPs at decision points during the development of the optic chiasm in Xenopus laevi [32].

The exact mechanism by which MMP-2 exerts Sema3A growth promoting effect needs further investigation. We failed to detect MMP-dependent receptor shedding to ensure the differential effect of Sema3A in axon and dendrites. This mechanism is considered as a very potent post-translational mechanism regulating membrane receptors [33]–[35] and as already been described for ADAMs, a subfamily of MMPs [33]–[35]. To better understand the functional link between semaphorin and MMP-2 we also searched for some steps of the signalling pathways involved. Several papers have described a positive link between PKC activity and MMP-2 expression and activity in various cell types [36]–[40]. Moreover, PKC signalling has been shown to inhibit dendritic outgrowth in cerebellar Purkinje cells [41]. The same group confirmed and extended this result; using KO-mice, they showed the specific importance of PKCα and PKCγ in the control of dendritic development [42]–[44]. The group of Inagaki demonstrated the existence of a calcium-dependent pathway for Sema3E (mouse semaphorin H) [45]. This semaphorin is structurally very similar to Sema3A. It acts as a chemorepulsive factor on sensory neurites [46], [47] and triggers neurite outgrowth in PC12 cells through an increase of the intracellular calcium level [45], [48]. More recently, the same group highlighted a similar signalling pathway for another semaphorin, Sema4D [49]. In this study, they described a PKC and calcium-dependent synergistic neurotrophic effect between Sema4D and NGF in PC12 cells. Finally, the group of Pfenninger has demonstrated that Sema3A-induced growth cone detachment and collapse require activation of PKC epsilon [50]. Our results show that the addition of the general PKC blocker Gö6976 is sufficient to abolish the Sema3A growth promoting effect and the increase of MMP-2 protein level and activity without change at the transcriptional level. Thus, a PKC-dependent pathway is activated upon Sema3A binding to recruit MMP-2 and ensure dendrite outgrowth. Considering the concentration of Gö6976 in our experiment (2.3 nM), this PKC dependent mechanism should be mainly due to PKCα isoenzyme [51], [52].

In conclusion, we provide additional results implicating MMPs in cortical development. We confirmed the functional link between MMPs and Semaphorins by identifying MMP-2 as an effective partner of Sema3A to trigger cortical dendrite outgrowth. This functional growth promoting effect probably requires a PKCα-dependent signalling pathway.

## Materials and Methods

### Cell culture

Cortical neurons were prepared from E15 mouse embryos as previously described [53]. Cerebral hemispheres were dissected in cold GBSS-glucose in order to isolate blocks of neocortex. They were placed in 5 ml of a solution of HBSS/glucose +0.25% trypsin, for 20 minutes, under agitation at 37°C. A volume of 5 ml of culture medium with serum containing 2 mM L-glutamine, 1 mg/ml glucose, 0.5 unit/ml penicillin, 0.5 μg/ml streptomycin, 10% foetal bovine serum (FBS) in DMEM (Dulbecco's modified Eagle medium, Gibco), was added to stop the activity of trypsin. The cells were centrifuged 5 minutes at 1000 g and the pellet was recovered in 1 ml of culture medium with serum. The cells were mechanically dissociated using a Gilson micropipette, filtered through 48 μm diameter pores and centrifuged again. A cellular counting was carried out and 2.10^5^ cells were seeded on glass coverslips covered with a substrate of poly-L-lysine (100 μg/ml). After 12 h in culture, the culture medium was replaced with fresh serum-free medium (2 mM L-glutamine, 1 mg/ml glucose, 0.5 unit/ml penicillin, 0.5 μg/ml streptomycin, 16 μg/ml putrescein, 52 ng/ml sodium selenite, 10 μg/ml transferrin, 5 μg/ml insulin, 3 ng/ml progesterone in DMEM). At this stage, neurons were treated with pharmacological inhibitors of MMPs (the large spectrum inhibitor of MMPs: GM6001, Calbiochem; MMP-2/MMP-9 Inhibitor I, Calbiochem) or with Sema3A±inhibitors. In another set of experiments, neurons were treated with function blocking antibodies±Sema3A. Neurons were incubated with 1 μg/ml anti-NRP1 polyclonal antibody obtained using a synthetic 14 amino acid peptide from the MAM domain of NRP1 [54], [55]. We identified axonal and dendritic processes by immunostaining of phosphorylated neurofilaments (SMI312, Steinberger Monoclonal, USA) and/or MAP2 respectively (see immunocytochemistry section). Axonal and dendritic length was determined using ImageJ software implemented with the NeuronJ plugin. Purified Sema3A was obtained as previously described [54]. Statistical analysis was performed using student's T test. Data are presented as mean +/− standard errors. The number of experiments and/or number of analyzed neurons is mentioned in the corresponding text.

### Histology and immunocytochemistry

E15 mouse brains were fixed at 4°C for 24 h in a 4% formaldehyde solution balanced with phosphate-buffered saline (PBS: 140 mM NaCl+9 mM Na_2_HPO_4,_ 2H_2_O+1.3 mM Na_2_PO_4_ H_2_O) and 20% sucrose. Fast freezing of the brains by direct immersion in −45°C isopentane was carried out to prepare sections for *in situ* zymography and double immunostainings. These sections were prepared using a cryostat (Reichert-Jung, LEICA). Slices were blocked in 3%-BSA-TBS for 30 minutes at 37°C, before overnight incubation at 4°C with primary antibodies anti-MMP-2 or -9 from Chemicon International, Hampshire, UK, ref. AB809 and ref. AB805 respectively, and/or anti-microtubule-associated protein 2 (MAP2) from Sigma, Saint Quentin, France, Ref. M4403, diluted in 3%-BSA-TBS, as a marker of dendrites. MMP-2 or -9 and MAP2 signals were revealed using a secondary antibody coupled with the avidin-biotin-fluorochrome Cy3 (Biosys, Compiègne, France, ref. BI 4807 and Jackson ImmunoResearch Laboratories, Suffolk, UK, ref. 016-160-084) or a secondary antibody coupled with Alexa 488 (Molecular Probes, Eugene, Oregon, ref. A-11017) respectively. Images were collected using a confocal microscope (LSM510, Zeiss, Le Pecq, France). Fluorescence profiles point out the presence or absence of co-localization between the two markers in the dendrites. The arrow shows a typical region across which line profiles were determined. While not quantitative this representation helps to confirm visual impressions of double staining.

### *In situ* zymography

To determine the enzymatic activity of MMPs *in situ*, we applied an adapted version (Gonthier et al., 2006) of the original method described by Oh and collaborators [56] . We used fluorescein-conjugated gelatin (from Enzcheck Assay kit, Molecular Probes, ref. E_12055) as a substrate of gelatinases MMP-2/-9. *In situ* zymography was carried out on E15 mouse brain sections (30 μm thickness) prepared with a cryostat (Reichert-Jung, LEICA). The sections were circled with a Dako Pen (DAKO) and incubated for 5 minutes with a reaction buffer (50 mM Tris-HCl+0.15 M NaCl+5 mM CaCl_2_+0.2 mM sodium azide; pH 7.6). Reaction mix consisting of 50 μl of reaction buffer +50 μg/ml of substrate (FITC-gelatin) was added to the section for 24 h in a wet darkroom, at 37°C. Negative control was obtained by the addition of 50 μM 1,10- phenantrolin to the reaction solution. We also used a potent inhibitor of MMP-2/-9 (Calbiochem, MMP-2/MMP-9 Inhibitor I, from VWR INTERNATIONAL S.A.S, Fontenay sous Bois, France) at the final concentration of 1 μM. Sections were mounted in Aqua Polymount (Poly Sciences, Eppelheim, Germany) without washes. The observations were performed with an inverted optical microscope (Axiovert 200M, Zeiss). The quantification of the fluorescence was determined using Metaview (Universal Imaging Corporation, Evry, France). In some experiments, *in situ* zymography was combined to immunohistochemistry. In this case, the zymographic reaction was performed during primary antibody incubation (using anti-MAP2 antibody, see details above), by addition of appropriate buffers.

### Western blot analysis of MMP expression

Western blots were performed on fresh conditioned culture media obtained from E15 dissociated cultured neurons. The different fractions were probed with the same antibodies used for immunohistochemistry (Chemicon International, ref. AB809 and AB805). Samples migrated in 8% polyacrylamide gels for 2 h at 25 mA before transfer on a nitrocellulose membrane (PROTAN, Perkin Elmer Life Sciences, ref. NBA085C) for 1 h at 250 mA. Membranes were blocked in 5%-BSA-TBS for 30 min and incubated with the primary antibody (dilution 1∶5000 for MMP-2 and 1∶20000 for MMP-9) overnight at 4°C under agitation. After several washes with TBS, the membranes were incubated with the secondary antibody F(ab')2 anti IgG (H+L) rabbit H.R.P. (P.A.R.I.S., ref. BI 4407) at a dilution of 1∶2000 in TBS for 1 h at room temperature. After washing in TBS, the blots were revealed with SuperSignal West Dura Extended Duration Substrate (Pierce, Rockford, Illinois, ref. 34076). Protein concentration was determined in the different samples by the Bradford method. Equal loading was verified by red Ponceau staining of the proteins in the absence of known referenced protein secreted at a constant concentration in the culture medium.

### RNA preparation and RT–PCR

Total RNA was extracted from freshly dissected E15 brain, E15 cortex and dissociated E15 cortical cell after 2 DIV. Dissociated cortical neurons were seeded in 10-cm tissue culture dishes (Becton Dickinson) covered with poly-L-lysine (100 μg/ml) at a concentration of 5×10^6^ cells/box. After 12 h in culture, the culture medium was changed with fresh serum-free medium (2 mM L-glutamine, 1 mg/ml glucose, 0.5 unit/ml penicillin, and 2% B27 supplement from Invitrogen), and cells were treated or not with Sema3A (100 ng/ml). RNA from the tissues and dissociated cells was extracted by using Qiagen RNeasy Mini Kit. Genomic DNA was eliminated by on-column DNase digestion according to supplier recommendations. RNA concentration and quality were assessed by spectrophotometry at 260 and 280 nm. cDNA was generated using Super-Script III reverse transcriptase first strand cDNA synthesis kit (Invitrogen). Briefly, 1 μg of total RNA was reverse-transcribed using random primers and reverse transcriptase (Invitrogen) and exposing the samples to 65°C for 5 min, followed by 42°C for 60 min and 75°C for 15 min. Of the resulting cDNA, 1 μl was used for transcript-specific PCR with Taq DNA polymerase (Taq Core Kit, Q-Biogene). Primer sequences used for detection of MMP2, MMP9 and GAPDH were 5′-CCGTGGATGATGCTTTTGCTCG-3′/5′-TCGTAGTTGGTTGTGGTCGC-3′, 5′-GGACAGCCAGACACTAAAGG-3′/5′-AGGAAGACGAAGGGGAAGAC-3′ 5′-CGGTGCTGAGTATGTCGTG-3′/5′-GTTATTATGGGGGTCTGGGATG-3′ respectively. 5′-GAGGGAGCAGGATTAGAGTC-3′/5′-AGAGAGGCAGTCAGTAGTTTGGG-3′ for Sema3A. PCR was performed incubating samples for 3 min at 94°C and then cycling 35 times for 30 s at 94°C, 30 s at 58°C, and 1 min at 72°C, followed by final elongation at 72°C for 5 min. Expression of GAPDH was used as PCR amplification positive control. Negative control for amplification consisted of RNA processed without the addition of reverse transcriptase. PCR products were subjected to electrophoresis on 1% TAE agarose gels containing ethidium bromide and visualized with UV light.

### MMP ELISA activity assays

Specific MMP enzymatic activities were determined as previously described [6], [57] in conditioned media using the Matrix Metalloproteinase Biotrak Activity Assay System for MMP-2 and -9 (Amersham Biosciences, Buckinghamshire, UK, ref. RPN2631 and ref. RPN2634 respectively). The whole procedure to measure the total activity of MMPs was performed according to manufacturer's instructions. Briefly, Elisa plates were incubated with culture media overnight at 4°C. The plates were then washed and incubated with a reaction solution containing a specific substrate of the matching MMP at 37°C and APMA (p-Aminophenylmercuric acetate) to activate all the pro-MMPs.

## Supporting Information

Figure S1 (0.14 MB TIF)
