# Supplementary figures and images for: A PKC-Dependent Recruitment of MMP-2 Controls Semaphorin-3A Growth-Promoting Effect in Cortical Dendrites
Source: PLoS One. 2009 Apr 8;4(4):e5099. doi: 10.1371/journal.pone.0005099 (PMC2663036; doi:10.1371/journal.pone.0005099)

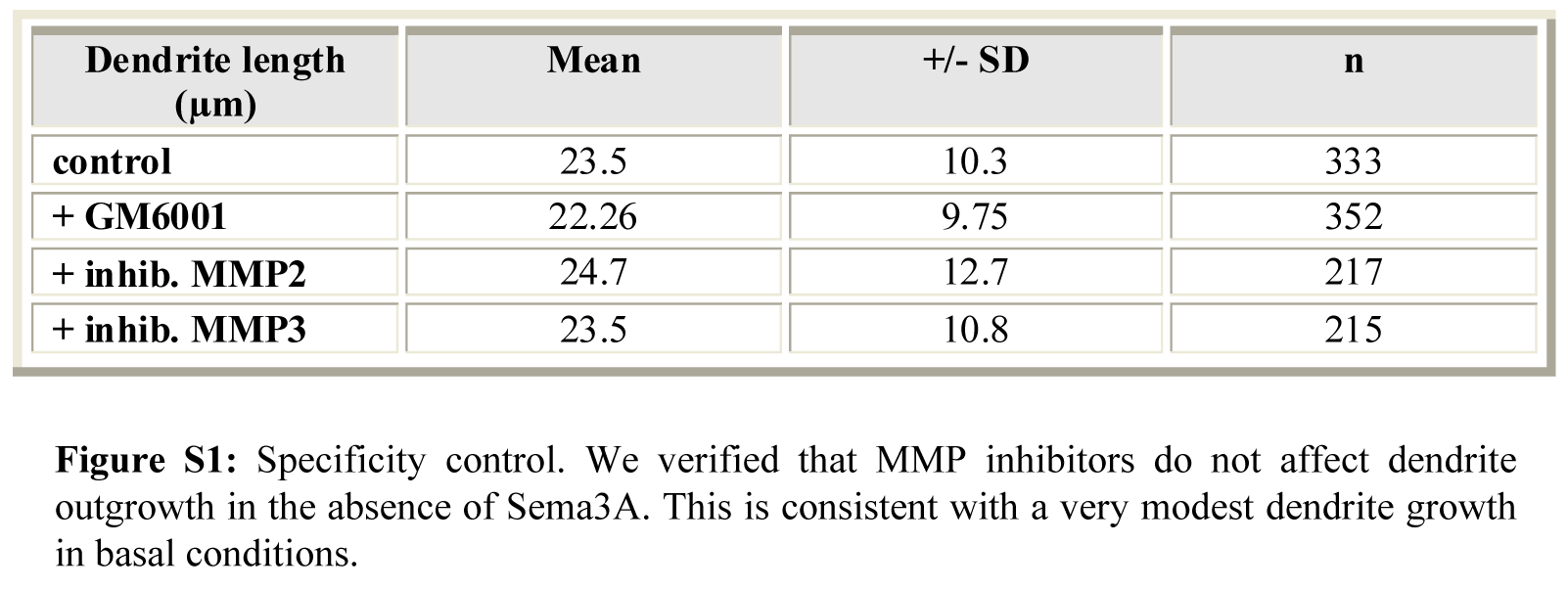

Supplement: Figure S1 — (0.14 MB TIF) [file pone.0005099.s001.tif]
